# Adverse childhood experiences and deviant peer affiliation among Chinese delinquent adolescents: the role of relative deprivation and age

**DOI:** 10.3389/fpsyg.2024.1374932

**Published:** 2024-06-06

**Authors:** Yuepeng Wang, Weijie Meng

**Affiliations:** ^1^School of Educational Science, Ludong University, Yantai, China; ^2^Institute for Education and Treatment of Problematic Youth, Ludong University, Yantai, China

**Keywords:** delinquent adolescent, adverse childhood experiences, deviant peer affiliation, relative deprivation, age

## Abstract

**Background:**

Deviant peer affiliation is considered a potential risk factor for adolescent delinquency. Due to the serious situation of adolescent delinquency in China, it is necessary to investigate the mechanisms by which adolescents associate with deviant peers.

**Objectives:**

The purpose of this study was to examine the association between adverse childhood experiences (ACEs) and deviant peer affiliation, the mediating effect of relative deprivation, and the moderating effect of age in a sample of Chinese delinquent adolescents.

**Methods:**

Five hundred and forty-two Special School students aged 11–18 years were interviewed and completed questionnaires, including demographics, adverse childhood experiences, deviant peer affiliation, and relative deprivation.

**Results:**

(1) After controlling for gender, adverse childhood experiences and deviant peer affiliation were significantly and positively associated among delinquent adolescents. (2) The effect of ACEs on deviant peer affiliation was mediated by relative deprivation. (3) Age played a moderating role not only in the relationship between ACEs and relative deprivation, but also in the indirect relationship in which ACEs influence deviant peer affiliation through relative deprivation; specifically, the indirect effect of ACEs influencing deviant peer affiliation through relative deprivation was stronger in early adolescence compared with late adolescence.

**Conclusion:**

Overall, early ACEs play an important role in deviant peer affiliation among delinquent adolescents and relative deprivation is an important mediating variable. The results of the present study emphasize the importance of cognitive interventions for delinquent adolescents who experience ACEs in early adolescence, which may be instructive for the prevention of adolescent delinquency.

## 1 Introduction

As a serious and emerging problem, adolescent delinquency in China is undoubtedly a cause for concern (Weng et al., 2016; Chang et al., 2021). According to *the White Paper on Juvenile Delinquency 2022* released by the Supreme People’s Procuratorate (SPP), the number of minors under the age of 18 committing crimes in China is on the rise (Supreme People’s Procuratorate, 2023). Gang involvement stands out as a distinctive characteristic of adolescent delinquency in China. Survey data gathered from Chinese youth reveal a striking parallel to findings in Europe and the United States, showing that individuals with a history of gang membership report a delinquency rate three times higher than their non-gang counterparts (Pyrooz and Decker, 2013; Weng et al., 2016). A meta-analysis based on risk factors for adolescent delinquency showed that antisocial peer associations were one of the “big four” risk factors and a strong predictor of adolescent delinquency (Andrews and Bonta, 2015). Consequently, in order to prevent deviant peer affiliation among Chinese adolescents, it is imperative to comprehend the possible risk factors influencing this behavior.

Adolescents are undergoing a second process of separation-individuation, during which they prefer to individuate away from their family while becoming more sensitive to peer pressure (Blos, 1967; De Looze et al., 2012; Yoon et al., 2019). Extensive research has documented that peers and friends have a significant influence on adolescents’ attitudes and behaviors (McGloin and Thomas, 2019; Portt et al., 2020). In peer relationships, deviant peer affiliation has been identified as a critical risk factor for risky behaviors (Zhu et al., 2016; Hinnant et al., 2019; Yang et al., 2023). However, a potential shortcoming of previous research is the lack of empirical data on high-risk youth (e.g., adolescent delinquents), as many empirical studies have been conducted with general samples (Li et al., 2013; Gao et al., 2022). According to Moffitt’s theory on the study of antisocial behavior, the non-delinquent group may consist of adolescents with limited antisocial behavior during adolescence, while the delinquent group may consist of adolescents who continue to delinquent behaviors over the life-course (Moffitt, 2005). The results of the empirical study also showed that deviant peer affiliation differed in the delinquent and normal samples, so the authors emphasized that the results of the study based on the normal sample could not be generalized to the convicted delinquent adolescents (Asscher et al., 2014; Sapouna, 2017). Furthermore, interventions based on general population data may not be effective for specialized populations (Tolan et al., 2008). To address this gap, the main objective of this study was to investigate the factors that contribute to an increasing or decreasing level of deviant peer affiliation among adolescents in at-risk groups.

The mechanisms of adolescent peer group formation suggest that they prefer to associate with groups that are homogeneous or similar (Richmond et al., 2019). However, as adolescents, their choices can also be limited by circumstance, especially when they encounter greater neighborhood disadvantage, dysfunctional schools, or parents with insufficient supervisory capacity (Zimmerman and Messner, 2011; Kalmakis and Chandler, 2014; Trinidad, 2021). Furthermore, these childhood experiences can also influence the behavioral choices of individuals in adolescence (Ng-Mak et al., 2002). Despite research suggesting that adverse childhood experiences are associated with deviant peer affiliation, the mediating and moderating mechanisms behind such relationships have received insufficient attention (Turanovic, 2019; Yoon et al., 2019; Jones et al., 2023). Therefore, this study investigated the relationship between adverse childhood experiences and deviant peer affiliation among delinquent adolescents and extended existing research by examining the mediating role of relative deprivation, as well as the moderating role of age on this mediation process.

## 2 Literature review

General strain theory proposes that when individuals experience strain or stress, they sometimes choose to engage in deviant or other behaviors to defuse it. Three sources of strain are defined: failure to reach positively valued goals, the loss of positive stimuli, and the presentation of negative stimuli (Andrews and Bonta, 2015), and one of these stressors is adverse childhood experiences. As an important predictor of adolescent delinquency, general strain theory provides a solid foundation for the antecedents of deviant peer affiliation, thus providing us with an explanatory framework. Agnew’s theory suggests that stress, including long-term chronic stress, may weaken attachment to significant others (family), adherence to traditional beliefs (that crime is wrong), and commitment to traditional activities (getting an education) as well as traditional institutions (school), which in turn increases the likelihood that adolescents will encounter criminal gangs. Furthermore, adverse childhood experiences may lead to peer rejection, which further increases individual pressure to join gangs for peer support (De La Rue and Espelage, 2014; Jones et al., 2023). The present study aims to explore potential mechanisms that mediate and moderate the effects of adverse childhood experiences on adolescents’ deviant peer affiliation.

### 2.1 Adverse childhood experiences and deviant peer affiliation

Adverse childhood experiences (ACEs) are a series of potentially traumatic experiences that occur before the age of 18 (Sosnowski et al., 2021), including harms that directly affect children (e.g., abuse and neglect, or extrafamilial violence) and indirectly affect children’s life circumstances (e.g., family dysfunction, or parental absence) (Hughes et al., 2017). The cumulative risk perspective emphasizes the importance of the number of ACEs, stressing that the greater the number of adversities experienced by an individual, the greater the negative impacts (Evans et al., 2013). Deviant peer affiliation is conceptualized as selective participation in deviant friendships while peer interaction and encouragement of criminal behaviors occur (Bao et al., 2022). Numerous empirical studies have shown that adverse childhood experiences are associated with negative life outcomes, such as a high risk of developmental, social, or behavioral delays (Cprek et al., 2020), substance abuse behaviors (Forster et al., 2018), and externalizing problem behaviors (Hunt et al., 2017). Similarly, the overwhelming correlation between deviant peer affiliation and externalizing problem behaviors has been confirmed (Zimmerman and Messner, 2011). Despite this, there has been relatively little research on how adverse childhood experiences affect delinquent peer affiliation (Jones et al., 2023). One of the few studies conducted by Trinidad demonstrated that individuals who experience family adversity during childhood were more likely to engage in deviant peer affiliation during adolescence and early adulthood (Trinidad, 2021). Jones et al. (2023) suggested that as children’s exposure to ACEs increases (by age 5), the likelihood of reporting association with delinquent peers increases.

In summary, there is some evidence to suggest a possible pattern between adverse childhood experiences and deviant peer affiliation. Most of the current research is conducted in general population groups, and it is worth considering whether their patterns of peer interactions are fully consistent with those of at-risk groups.

### 2.2 The mediating role of relative deprivation

Relative deprivation is a judgment that an individual is worse off compared to certain standards, and is accompanied by feelings of anger and resentment (Smith et al., 2012; Li et al., 2023). According to the theoretical model of relative deprivation described by Smith and colleagues, relative deprivation involves three steps: cognitive comparisons, cognitive appraisals, and justice-related affect (Smith et al., 2012). During adolescence, individuals’ peer interactions increase, providing a basis for social comparisons (McGloin and Thomas, 2019). Adolescents often believe that they deserve positive childhood experiences and that they are not responsible for the occurrence of adverse childhood experiences. Consequently, those who have experienced adversity may perceive themselves as disadvantaged after cognitive appraisal, and develop a sense of relative deprivation, which in turn leads to behavioral consequences resulting from that sense of relative deprivation (Li et al., 2023). Therefore, it is reasonable to expect that feelings of relative deprivation play a mediating role in ACEs and deviant peer affiliation interactions. Adolescents who experience more ACEs may develop higher levels of relative deprivation, which leads to more deviant peer affiliation.

There are two reasons to support the argument for relative deprivation playing a mediating role. Firstly, adolescents who experience ACEs may experience higher levels of relative deprivation. While there is no direct evidence linking ACEs to feelings of relative deprivation, there has been a significant body of research demonstrating that individuals who experience ACEs feel higher levels of shame, more envy, and are more susceptible to psychiatric disorders such as depression and anxiety than controls, as well as having stronger motivation to make social comparisons (Dehon and Weems, 2010; Xiang et al., 2018, 2020). Meanwhile, comparisons with others are a key mechanism in the creation of relative deprivation (Smith et al., 2012). Furthermore, some studies have demonstrated that individuals who lacked social support or experienced childhood maltreatment reported higher levels of relative deprivation (Zhang and Tao, 2013; Li et al., 2023). Second, relative deprivation may be positively correlated with deviant peer affiliation. Relative deprivation may lead to negative outcomes such as anger or aberrant behavior in individuals. Research based on the Chinese context suggested that relative deprivation has a positive effect on violent crime (Smith et al., 2012; Song et al., 2022). Through three experimental studies, researchers have shown that participants in a relative deprivation condition exhibit higher levels of aggression than individuals who get along in a relative contentment condition (Greitemeyer and Sagioglou, 2017). Similarly, it has been demonstrated that a sense of relative deprivation is significantly and positively associated with deviant peer affiliation (Li et al., 2023).

### 2.3 The moderating role of age

Although ACE is an important predictor of relative deprivation, it is crucial to consider the impact of adolescents’ cognitive development at different stages, as this is a process that develops over time (Callan et al., 2015). The theory of relative deprivation posits that an individual’s sense of relative deprivation involves three integral parts: comparing oneself to others, recognizing that one is disadvantaged, and experiencing negative emotions (Smith et al., 2012). Perspectives from developmental psychology suggest that adolescence is a period of active social comparison (Harter, 2015). Firstly, upon entering secondary school, students are exposed to more information regarding social comparison due to a more diverse peer group and a developmental stage that places greater emphasis on peers rather than family (Yoon et al., 2019; Huang and Zhu, 2020). Secondly, many studies have pointed out that the transition to secondary school is marked by a shift in educational emphasis, which is reflected in teachers’ greater focus on social comparisons of students, such as publishing grades (Fredricks and Eccles, 2002; Gürel et al., 2020). At the same time, research has shown that self-evaluation fluctuates during early adolescence (Siffert et al., 2012). Meta-studies have indicated that the stability of self-evaluation increases throughout adolescence and adulthood (Trzesniewski et al., 2003), with one possible explanation being changes in significant others during early adolescence (Siffert et al., 2012). In addition, evidence from experimental studies suggested that there is an increase in adolescents’ ability to regulate their emotions from early to mid-adolescence (Theurel and Gentaz, 2018). Therefore, it is reasonable to hypothesize that age is a moderating variable for the relative deprivation of ACEs. According to the researcher’s recommendations, adolescents can be categorized into early adolescence (age 10–14 years) and late adolescence (age 15–19 years) based on their age (Irwin et al., 2002). In early adolescence, ACEs were stronger predictors of relative deprivation; in late adolescence, ACEs predicted relative poverty weakly. Different ages of adolescents may influence not only the relationship between ACEs and relative deprivation, but also the indirect role of ACEs in influencing deviant peer affiliation through relative deprivation. Specifically, the indirect effect of ACEs influencing deviant peer affiliation through relative deprivation was stronger in early adolescence; it was weaker in late adolescence.

**Figure 1:**
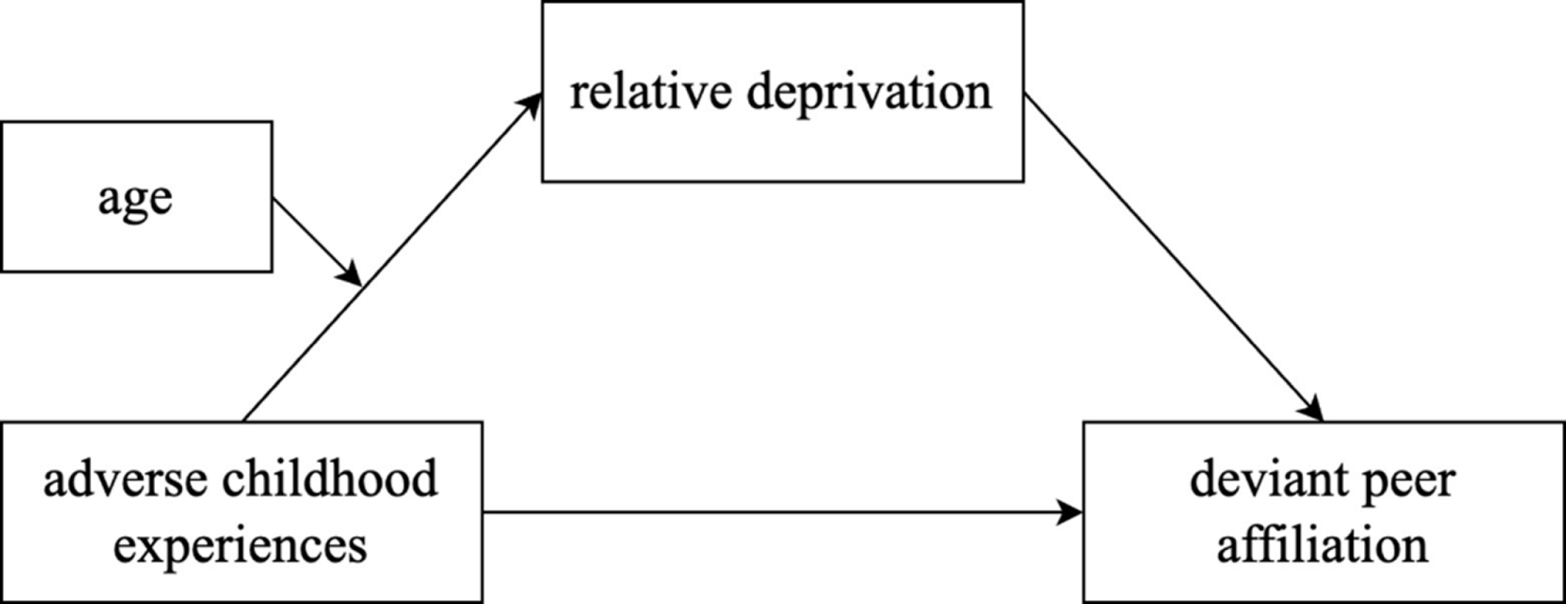
The moderated mediation model.

Thus, this study aims to examine the mediating roles of relative deprivation between ACEs and deviant peer affiliation, as well as the moderating role of age in this process, using a sample of delinquent adolescents in China (see Figure 1).

### 2.4 Research hypotheses

Hypothesis 1: ACEs are positively associated with deviant peer affiliation.

Hypothesis 2: Relative deprivation mediates ACEs and deviant peer affiliation. Specifically, ACEs predicted deviant peer affiliation via relative deprivation.

Hypothesis 3a: Age moderates the effect of ACEs on relative deprivation. In early adolescence, ACEs were stronger predictors of relative deprivation; in late adolescence, ACEs predicted relative poverty weakly.

Hypothesis 3b: Age moderates the indirect effects of ACEs influencing deviant peer affiliation through relative deprivation. The indirect effect of ACEs influencing deviant peer affiliation through relative deprivation was stronger in early adolescence; it was weaker in late adolescence.

## 3 Method

### 3.1 Participants

The study sample consisted of 542 adolescents from five Specialized Schools in China. In China, as stipulated in the Law on the Prevention of Juvenile Delinquency, specialized schools are designed for three categories of persons: minors who have committed criminal acts, minors who have committed serious violations of public order, and minors who have committed general violations of public order (Leng and Jiao, 2023; Zhang and Yao, 2023). Participants ranged in age from 11 to 18, with an average of 14.50 years (*SD* = 1.36). Among them, 78.20% (424/542) were males, 21.8% (118/542) were females; 17.70% have no siblings. 50.20% (272/542) could be classified as early adolescence and 49.80% (270/542) as late adolescence.

### 3.2 Measures

#### 3.2.1 ACEs

This study used the Revised Adverse Childhood Experiences-International Questionnaire (ACE-IQ) to assess adolescents’ adverse childhood experiences (Zhou et al., 2022). The 14-item scale consists of 22 descriptions of childhood experiences and 4 factors: childhood abuse and neglect, extrafamilial violence, family dysfunction, and parental absence (e.g., “Your parents, guardians or other family members have threatened to abandon you or kick you out of the house.”). The questionnaire showed good test–retest reliability (0.73) and K-R20 coefficient (0.71) in the Chinese adolescent population (Zhou et al., 2022). Responses were coded as 1 = yes, 0 = no; a cumulative score was created by summing the items, with possible scores ranging from 0 to 14 ACEs. For the current study, its K-R20 coefficient was 0.69.

#### 3.2.2 Deviant peer affiliation

This study measured adolescents’ deviant peer affiliation status using the Deviant Peer Affiliation Scale, which consists of eight items (Li et al., 2016). Adolescents were asked to rate on a five-point scale the percentage of peers who had engaged in transgressive behaviors in the past 12 months (e.g., truancy or skip school, steal things); ranging from 1 (none) to 5 (all). Response scores for the eight items were averaged, with higher scores representing more deviant peer affiliation. The scale has been widely used among Chinese subjects and has shown good reliability and validity (Li et al., 2023). In this research, its Cronbach’s *α* was 0.79.

#### 3.2.3 Relative deprivation

The four-item Relative Deprivation Scale measured individuals’ subjective feelings of relative deprivation (Ma, 2012). Participants responded to each item on a 5-point scale ranging from 1 (strongly disagree) to 5 (strongly agree), with higher scores indicating stronger relative deprivation felt by the individual. An example item is “Compared to the people around me, I’m at a disadvantage in every aspect of my life, my studies, etc.” The scale has been widely used among Chinese subjects and has demonstrated good reliability and validity (Zhao and Zhang, 2022). In this research, its Cronbach’s *α* was 0.72.

### 3.3 Procedure

The current study was approved by the Research Ethics Committee of the first author’s university. Data collection was conducted by the first author of this study. Respondents voluntarily participated in an anonymous questionnaire, and informed consent was obtained from the participants prior to commencement. Participants were informed of their right to refuse participation and to withdraw. The first author of this paper entered the classroom as a researcher after obtaining permission from the school, and the participants filled out the questionnaire in the classroom accompanied by the classroom teacher.

### 3.4 Data analysis

All data were dealt with in SPSS 27.0. We handled the missing data by mean imputation. The skewness and kurtosis of ACEs, deviant peer affiliation and relative deprivation were 0.10 and − 0.55, 0.11 and − 0.16, 0.03 and 0.08, which fell within the acceptable range (*Sk* < |2.0| and *Ku* < |7.0|; Mueller et al., 2010). The results of common method bias showed that total eigenvalues >1 were obtained without rotation. Additionally, there were 21 common factors, and the explanation rate of the first common factor was 13.41%, which was lower than 40%. Therefore, there was no serious common method deviation.

Then, the data were analyzed in three steps. First, we conducted descriptive statistics and analyzed the correlations between the variables. We also analyzed the prevalence of ACEs and statistics on deviant peer affiliation. Second, Hayes’s (2013) PROCESS 3.5 macro (Model 4) was used to examine whether relative deprivation mediated the relationship between ACEs and deviant peer affiliation. Meanwhile, the study used 5,000 bootstrap samples and corrected for bias according to 95% confidence intervals (CIs) to calculate the indirect effects of each variable. If the CI for the indirect effect did not include zero, it indicated that the indirect effect was significant at *p* = 0.05 (Shrout and Bolger, 2002). Third, we explored whether this mediation process was moderated by age using Hayes’s (2013) PROCESS 3.5 macro (Model 7). In all analyses, we controlled for gender as a covariates.

## 4 Results

### 4.1 Preliminary analyses

Firstly, 67.50% (366/542) of these 542 adolescents had the experience of being left-behind. Secondly, we tested the incidence of ACEs, the average number of ACEs experienced by adolescents was 5.07 ± 2.73 in early adolescence and 5.57 ± 2.55 in late adolescence. 97.4% (528/542) of adolescents reported one or more types of ACEs, of these, 85.8% (465/542) reported experiencing childhood abuse and neglect, 79.3% (430/542) reported experiencing extrafamilial violence, 29.5% (160/542) reported experiencing family dysfunction, and 79.0% (428/542) reported experiencing the absenteeism of a father or mother. Additionally, 99.8% (541/542) of adolescents reported one or more types of deviant peer affiliation.

Means, SDs, and correlation matrix of variables were presented in Table 1. Overall, all results were consistent with our expectations. Specifically, ACEs were positively correlated with relative deprivation and deviant peer affiliation. Furthermore, relative deprivation was positively correlated with deviant peer affiliation.

**Table 1:** Descriptive statistics and correlations among variables.

	*M*	*SD*	1	2	3	4	5
1. Gender	1.22	0.41	1				
2. Age	14.50	1.36	−0.15^**^	1			
3. ACEs	5.32	2.65	0.08	0.10^**^	1		
4. Deviant peer affiliation	2.84	0.71	0.00	0.01	0.27^**^	1	
5. Relative deprivation	11.89	2.83	0.03	−0.06	0.25^**^	0.30^**^	1

^**^p < 0.01. Gender was dummy coded such that 1 = boys and 2 = girls.

### 4.2 Testing for mediation effect

As presented in Table 2, the results showed that after controlling for gender, ACEs were positively correlated with deviant peer affiliation (*b* = 0.07, *p* < 0.001), ACEs were positively correlated with relative deprivation (*b* = 0.27, *p* < 0.001), and relative deprivation was positively correlated with deviant peer affiliation (*b* = 0.06, *p* < 0.001), thus, supporting Hypothesis 1. When relative deprivation was introduced as a mediating variable, the relationship between ACEs and deviant peer affiliation remained significant (*β* = 0.06, *p* < 0.001). Thus, relative deprivation partially mediated the relationship between ACEs and deviant peer affiliation. Furthermore, this study calculated 95% CI based on a 5,000 bootstrap resampling to further test the indirect effect, the results showed ACEs increased deviant peer affiliation through relative deprivation (*b* = 0.02, 95%CI [0.01, 0.03]), supporting Hypothesis 2.

**Table 2:** Testing the mediation effect.

The regression equation		Overall fitting index		Significance of regression coefficient			
Outcome variable	Predictors	*R* ^2^	*F*	*b*	Boot LLCI	Boot ULCI	*t*
Deviant peer affiliation	Gender	0.07	21.62^***^	−0.03	−0.18	0.11	−0.49
	ACEs			0.07	0.05	0.10	6.58^***^
Relative deprivation	Gender	0.06	18.47^***^	0.03	−0.53	0.60	0.12
	ACEs			0.27	0.18	0.36	6.05^***^
Deviant peer affiliation	Gender	0.13	27.15^***^	−0.04	−0.17	0.10	−0.53
	ACEs			0.06	0.03	0.08	5.06^***^
	Relative deprivation			0.06	0.04	0.08	5.95^***^

^***^p < 0.001.

### 4.3 Testing for moderated mediation

As Table 3 illustrates, ACEs positively predicted relative deprivation (*b* = 0.57, *p* < 0.001). The interaction effect of ACEs and relative deprivation on age was significant (*b* = −0.20, *p* < 0.05).

**Table 3:** Testing the moderated effect.

The regression equation		Overall fitting index		Significance of regression coefficient			
Outcome variable	Predictors	*R* ^2^	*F*	*b*	Boot LLCI	Boot ULCI	*t*
Relative deprivation	Gender	0.08	10.93^***^	−0.07	−0.63	0.5	−0.23
	ACEs			0.57	0.29	0.84	4.07^***^
	Age			−0.31	−0.77	0.16	−1.29
	ACEs × age			−0.20	−0.37	−0.02	−2.20^**^
Deviant peer affiliation	Gender	0.13	27.15^***^	−0.04	−0.17	0.1	−0.53
	ACEs			0.06	0.03	0.07	5.06^***^
	Relative deprivation			0.06	0.04	0.08	5.95^***^

^**^p < 0.01, ^***^p < 0.001.

**Figure 2:**
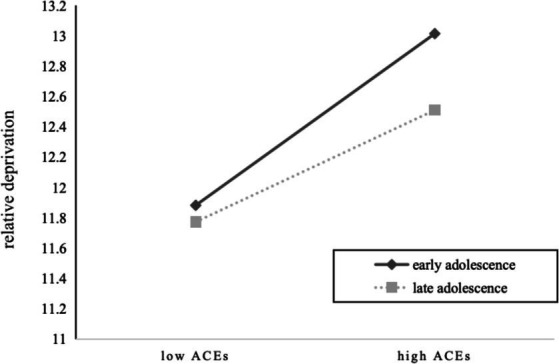
Moderating effect of Relative deprivation on the link between ACEs and deviant peer affiliation.

To further illustrate the effect of age on the interaction between ACEs and relative deprivation (see Figure 2), the study conducted a simple slope analysis. Simple slope tests showed that the ACEs were positively associated with relative deprivation for delinquent adolescents in early adolescence (*b_simple_* = 0.37, *t* = 6.01, *p* < 0.001). However, for those with late adolescence, the association was still significant but much weaker (*b_simple_* = 0.17, *t* = 2.61, *p* < 0.001). Thus, Hypothesis 3(a) was supported.

In addition, this study tested conditional indirect effects as suggested by Preacher et al. (2007), which focus on examining mediating effects that are significant at different levels of moderating variables. Findings showed that relative deprivation mediated stronger effects of ACEs on deviant peer affiliation in early adolescence (*b* = 0.02, 95%CI [0.01, 0.03]). In late adolescence, relative deprivation mediated weaker effects in ACEs on deviant peer affiliation (*b* = 0.01, 95%CI [0.01, 0.02]), supporting Hypothesis 3(b).

## 5 Discussion

The purpose of this study was to examine the link between ACEs and deviant peer affiliation among delinquent adolescents. A moderated mediation model was developed to test the mediating role of relative deprivation and the moderating role of age.

### 5.1 The prevalence of ACEs

The prevalence of adverse childhood experiences measured in this study was higher among the delinquent adolescents compared to other studies involving Chinese adolescents. For example, 89.4% of adolescents in a study based on 14,820 Chinese general adolescents reported one or more types of adverse childhood experiences (Wan et al., 2019). One possible explanation for this difference is the variation in subject groups between the two studies. The present study involved delinquent adolescents, while previous research has shown that juvenile violent offenders have experienced significantly more ACEs, including abuse, than non-offenders (Chang et al., 2021). Additionally, cultural differences in China must be considered to explain this variation (X. Li et al., 2014). On the one hand, “Seriousness and lack of warmth” is considered a worthwhile parenting style in China; on the other hand, many adolescents experienced parental absence and were cared for by non-parent guardians (usually grandparents), which increased their likelihood of exposure to ACEs (Zhou et al., 2020).

### 5.2 The influence of ACEs on deviant peer affiliation

The study found that the status of adolescents experiencing childhood adversity predicted deviant peer affiliation, which means that experiencing adversity may have an impact on peer affiliation and friendship choices, specifically the more adversity adolescents experience the more likely they are to engage in deviant peer affiliation, which supports Hypothesis 1. This result was consistent with the results obtained by previous researchers in the general samples (Yoon et al., 2019; Jones et al., 2023). ACEs can lead to disruptions in multiple developmental processes from a developmental psychopathology perspective. These disruptions can result in impaired development in multiple domains of functioning, which may make adolescents less able to resist negative life influences such as deviant peer affiliation (Fanti et al., 2018; Morrow and Villodas, 2018).

This can also be explained by the early adolescent upbringing. In China, left-behind children lack warmth and protection from their families and face many mental health issues, forcing them to seek peer support and interaction (Zhao et al., 2015; Yang, 2022). The factor of adolescent role models has to be considered. Adolescents who typically experience more ACEs live in disadvantaged neighborhoods or schools and may face high rates of delinquent peer role models, which may exacerbate the likelihood that they will engage in deviant peer affiliation (Baglivio et al., 2017). Family dysfunction may weaken the family’s supervisory function and fail to protect adolescents from deviant peers or gangs. Prolonged exposure to violence may cause adolescents to misinterpret social norms, affecting the individual’s value construction and leading to more deviant peer affiliation (Trinidad, 2021). Overall, the results of this study suggest that ACEs predicted deviant peer affiliation.

### 5.3 The mediating role of relative deprivation

The current study examined the mediating role of relative deprivation between adverse childhood experiences and deviant peer affiliation. The results confirmed Hypothesis 2. Specifically, ACEs may not only directly affect adolescents’ deviant peer affiliation, but may also lead to it by stimulating an individual’s sense of relative deprivation and thereby contributing to deviant peer affiliation. The results of this study also explain the phenomenon that not every individual who experiences childhood adversity engages in deviant peer affiliation. A possible explanation is that prolonged exposure to childhood adversity alters an individual’s perceptions (e.g., developing a sense of relative deprivation), leading to engagement in deviant peer affiliation. Scholars and clinicians believe that exposure to stressors (e.g., ACEs) early in the life course has direct effects on the immune, metabolic, and endocrine systems, and indirect effects through stress-related hormones that affect cognitive, social, and emotional development (Deighton et al., 2018; Jones et al., 2023). The theory of relative deprivation offers a rational explanation for this phenomenon. As adolescents’ peer affiliations expand and their individual cognition develops, they gradually recognize that they have experienced poverty and disadvantage in the past. Social comparisons then lead to a sense of unfairness or unmet needs. To rectify this, they may resort to shortcuts such as engaging with deviant peers (Yang et al., 2021; Li et al., 2023). In particular, researchers believe that the “envy effect” is widespread in China due to cultural and customary reasons, so that facing adversity or relative poverty leads to a stronger sense of relative deprivation, which in turn leads to transgressive behaviors or delinquency (Song et al., 2022).

In addition to the overall mediation model, each individual aspect of the mediation model deserves attention. Specifically, for the first component of the mediation process (i.e., ACEs→relative deprivation), the results of the present study indicated that ACEs were significantly and positively associated with relative deprivation. This is similar to previous findings such as that adolescents who reported parental neglect and estrangement believed that they deserved better treatment, and that individuals who had experienced childhood maltreatment report higher levels of individual relative deprivation (Rothman and Steil, 2012; Li et al., 2023). One possible explanation is that adolescents aspire to be treated as equals. However, through social comparisons, they may find themselves in a position of poverty and disadvantage, which can lead to a sense of relative deprivation. In the second part of the mediation process, this study found that relative deprivation was significantly and positively related to deviant peer affiliation. This finding is consistent with previous research indicating that individuals who experience greater relative deprivation are more likely to associate with deviant peers (Li et al., 2023). Self-determination theory may explain why individuals tend to satisfy their basic psychological needs. When negative experiences, such as relative deprivation, occur in real life, individuals may seek alternative ways to satisfy themselves, even if those ways are not recognized. For example, they may engage with deviant peers in order to gain higher peer status, more material wealth, or faster gratification (Lu et al., 2023). Simultaneously, research suggested that in adolescent antisocial peer groups, peer victimization, including aggression, is likely to be perceived as popular or “cool,” which may explain why adolescents choose deviant peer affiliation for self-gratification after experiencing relative deprivation (Chen et al., 2015).

### 5.4 The moderating role of age

Although studies have examined the effect of age differences on relative deprivation (Callan et al., 2015), few have focus on adolescents. Consistent with the hypothesis, the present study investigated the moderating role of age in the relationship between ACEs and relative deprivation among adolescents, thereby confirming that age moderates the indirect effects of ACEs on deviant peer affiliation through relative deprivation. Specifically, individuals who have experienced more ACEs in early adolescence tend to experience more relative deprivation. In contrast, this predictive effect diminishes in late adolescence. Childhood adversity, a toxic stressor, has been shown to impact brain development, cognitive performance, and self-regulation (McEwen and McEwen, 2017). Adolescents are in a developmental stage where they cope with stress and self-regulation. This may explain why adolescents at different ages differ in the impact of adverse childhood experiences and relative deprivation. Adolescents are not only individuals but also social beings. Changes in social comparisons can also affect individuals’ relative deprivation. During the initial stage of “separation-individualization,” early adolescents experience greater volatility compared to late adolescents who have a fixed peer group. This may be due to the fact that with the stabilization of social comparison factors and improved ability for cognitive self-regulation, the impact of ACEs on relative deprivation diminishes in late adolescence, and also predicts fewer deviant peer affiliations.

### 5.5 Limitations and future directions

As with all studies, there are several important caveats to keep in mind for this study. Firstly, despite the theoretical support for this study and the extensive support from prior empirical research, we cannot establish causal relationships between variables due to the limitations of the cross-sectional research methodology. Future studies should validate these results using longitudinal tests or intervention experiments. Secondly, the results were derived from subjects’ self-reports, and due to the social approval effect, some subjects may have concealed their true situation, affecting the accuracy of the results. For example, not all participants may have been willing to disclose their ACEs. Future researches should use various experimental designs to mitigate the impact of the social approval effect. Thirdly, the data in this study were collected exclusively from Specialized Schools in China, and there is a lack of comparative data from students in ordinary schools. Therefore, the generalization of the results of this study needs to be approached with caution. This is because previous studies have shown that at-risk adolescents differ from normal adolescents in their cognitive and behavioral styles (Chang et al., 2021). Finally, due to gender differences in juvenile delinquency rates, most students in specialized schools are male, which may affect the results.

### 5.6 Practical implications

From a practical point of view, this study is instructive for protective services for delinquent youth in China. First, this study focused on a neglected group. Previous studies have primarily focused on delinquent and ordinary adolescents, but less attention has been given to minor delinquent adolescents. The results of this study can enrich the understanding of minor delinquent adolescents. Second, this study found that the reporting rate of ACEs among delinquent adolescents reached 97.4%, a grim figure that deserves our attention. On the one hand, we have to consider the cultural context of China, where harsh parental discipline, the challenging situation of left-behind children, and the neglect of children’s emotions all affect the healthy growth of adolescents. On the other hand, it also provides guidance for child protection services. This reminds authorities to prioritize the protective upbringing of children. For instance, when families are unable to provide a suitable environment for their children, protective schools, such as China’s specialized schools, can offer a better alternative. Third, it is necessary to offer adolescents who experience ACEs positive peer interaction models to enhance their resilience. For example, there is a need for specialized developmental programs to promote adolescents’ social skills, social competence and positive friendship relationships. Finally, this study emphasized the differential impact of age, reminding us to focus on the critical period of adolescent development, such as scientifically guiding adolescents to make reasonable cognitive attributions at the beginning of their secondary school years. For instance, psychological interventions and cognitive training can be used to reduce the negative effects of ACEs.

## 6 Conclusion

This study aimed to explore the relationship between ACEs and deviant peer affiliation among Chinese delinquent adolescents, as well as the mediating role of relative deprivation and the moderating role of age. The study found that ACEs predicted relative deprivation more strongly in early than in late adolescence. Relative deprivation mediated the relationship between ACEs and deviant peer affiliation. Specifically, the indirect effect of ACEs influencing deviant peer affiliation through relative deprivation was stronger in early adolescence compared with late adolescence. This result serves as a reminder of the importance of focusing on the role of ACEs in future negative effects among delinquent adolescents, as well as on the cognitive processes by which they internalize adversity, especially in early adolescence.

## Data availability statement

The original contributions presented in the study are included in the article/Supplementary material, further inquiries can be directed to the corresponding author/s.

## Ethics statement

The studies involving humans were approved by Ludong University Ethics Committee. The studies were conducted in accordance with the local legislation and institutional requirements. Written informed consent for participation in this study was provided by the participants' legal guardians/next of kin.

## Author contributions

YW: Writing – original draft. WM: Writing – review & editing.
