# Supplementary material for: Adverse childhood experiences and deviant peer affiliation among Chinese delinquent adolescents: the role of relative deprivation and age
Source: Front Psychol. 2024 Jun 6;15:1374932. doi: 10.3389/fpsyg.2024.1374932 (PMC11187725; doi:10.3389/fpsyg.2024.1374932)
Supplement: Supplementary file 2 [file Table_1.DOCX]

Supplementary Material

## The Revised Adverse Childhood Experiences-International Questionnaire (ACE-IQ)

Please answer the following questions based on your own experiences and facts growing up. Please choose "Yes" or "No" to answer whether or not any of these situations have ever occurred in your life, and there is no right or wrong answer. Please note that for questions about "parents, guardians, or other family members," all three do not have to be present at the same time, and any one of them can answer "yes" to any of the situations described in the question.

- 1. You have been rebuked, cursed, insulted or humiliated by a parent, guardian or other family member in the home.
  2. Your parent, guardian or other family member has threatened to abandon you or throw you out of the home.

1. Your parent, guardian, or other family member slaps you, kicks you, punches you, or otherwise hits you.
   1. Someone forcibly touches or fondles you when you do not want to be touched or fondled.
   2. Someone made you touch or fondle someone else's body when you did not want to touch him/her.
   3. Someone forcing you to be naked or purposely exposing your body in front of you.
   4. When you don't want to have sex with someone, he/she tries to force it.
2. Your father, mother or guardian won't send you to school even if you have free time.
   1. Your father, mother or guardian does not provide you with enough food even when it is easy to do so.
   2. Your family members are often too busy with other things to take care of you.
3. In real life you have seen or heard family members or other people being yelled at, cursed, insulted/beaten, kicked/hit with objects such as sticks, bottles, knives, whips, etc.
4. You have witnessed or experienced fires, car accidents, earthquakes, drownings, suicides, or other events that have resulted in injury or death.
   1. You (have) been bullied or ostracized and isolated by peers.
   2. You (have) been bullied or attacked on social media (QQ, circle of friends, public number, Weibo, Shake, etc.) with verbal abuse, spoofing, threats, defamation, etc.
5. You (have) lived with a family member who has been in prison.
6. You (have) lived with a family member who suffers from depression, other mental illness, or suicidal tendencies.
7. You (have) lived with a family member who abuses alcohol, drugs or gambling.
   1. Growing up, you (have) lived apart from your father or mother for an extended period of time (more than six months).
   2. Your family is included in the urban or rural low income, poverty alleviation assistance program or has serious financial difficulties.
   3. While growing up, you have been placed in foster care to live with another family.
8. Your parents (have) separated or divorced.
9. Your father or mother is deceased.

## Deviant peer afﬁliation scale: 1 (none) to 5 (all).

During the past twelve months, how many of your close friends had engaged in the following behaviors:

1. Smoke cigarettes.
2. Drink alcohol.
3. Bully someone or threaten to hurt someone.
4. Cheat on exams.
5. Steal things.
6. Internet addiction.
7. Getting into trouble at school.
8. Truancy or skip school.

## Relative Deprivation Scale: 1(strongly disagree) to 5(strongly agree)

1. My life should be better than it is now for all the hard work and dedication I've put into it.
2. I always feel that other people have taken what is rightfully mine.
3. Compared to the people around me, I'm at a disadvantage in every aspect of my life, my studies, etc
4. Most of the rich people in our society got rich through dishonorable means.
